# Chimpanzees’ working memory is not affected by the presence and activity of zoo visitors

**DOI:** 10.1007/s10071-025-02014-4

**Published:** 2025-12-01

**Authors:** Aurélien Frick, Emma Suvi McEwen, Amanda M. Seed

**Affiliations:** School of Psychology and Neuroscience, University of St Andrews, St Mary’s Quad, St Andrews, KY16 9JP UK

**Keywords:** Visitors’ presence, Conspecifics’ presence, Chimpanzees, Working memory, Social presence

## Abstract

Cognitive research in non-human primates is increasingly conducted in zoos, where zoo visitors are likely to be present and observe the testing sessions. Previous zoo research has shown that such presence can modulate cognitive performance in monkeys, but similar investigations on great apes are still lacking. Here, we investigated the influence of the presence and activity level of zoo visitors on eight chimpanzees (*Pan troglodytes*) while they completed a working memory task with various difficulty levels. We also recorded whether their conspecifics were present or not in the testing area, and their approximate physical distance. We found that the presence and activity level of the visitors had no effect on chimpanzees’ working memory, regardless of the cognitive demands of the task. Similarly, the presence of conspecifics and how far they were from the individual being tested was not found to significantly influence performance either. These non-significant effects were supported by Bayesian analyses giving support for the null hypotheses. These results suggest that zoo-living chimpanzees are not distracted by visitors when completing a cognitive task. We suggest new directions to further explore social presence effects in great apes.

**Supplementary Information:**

The online version contains supplementary material available at 10.1007/s10071-025-02014-4.

## Introduction

Comparative cognitive research provides a unique opportunity to better appreciate the cognitive architecture of animal minds, but also to infer which human cognitive mechanisms are shared with other non-human species and which ones appeared more recently in our phylogenetic history (Durdevic and Call 2022). To this aim, the study of non-human primate (hereafter primate) cognition is essential and it has a long history in the field (e.g., Köhler 1921; Yerkes and Yerkes 1929). But most of what we currently know about primate cognition was discovered in the last forty years (e.g., Call and Tomasello 2024; Gentner 2007; Seed and Tomasello 2010). These major advances have been mainly driven by an increase of studies taking place in university research institutes but also in zoos. Indeed, in more recent years, zoos have been increasingly used for cognitive research as they allow access to a higher number of individuals taking part in cognitive research as well as a greater species diversity (Hopper 2017; McEwen et al. 2022). Consequently, much of the current cognitive research is being conducted in the presence and absence (hereafter presence) of zoo visitors as part of the overall zoo experience, and sometimes in the presence of the conspecifics. The present article investigates whether the cognitive performance of chimpanzees (*Pan troglodytes*) on a working memory task is influenced by the presence of visitors and conspecifics. The motivations for such investigation are twofold as it would provide valuable insights into theoretical conceptions of social presence effects from an evolutive perspective, and it would also contribute to improve research practices and comparability across different contexts.

Investigating the influence of the presence of social agents on cognitive performance is one of the first areas (Allport 1924; Triplett 1898) as well as one of the most intense fields of research in human psychology (for reviews, see Bond and Titus 1983; Garcia-Marques and Fernandes 2024; Guerin and Innes 1984; van Meurs et al. 2022). Interestingly, this question was also of interest for early comparative researchers, though not on cognitive performance but on food behaviours (e.g., Harlow and Yudin 1933; Ross and Ross 1949). It is only in the 60’s that a first cognitive study was conducted and showed that cognitive performance of rhesus monkeys (*Macaca mulatta*) was facilitated by the presence of conspecifics, though with some variations when hierarchal rank was considered. Indeed, the performance in the alone condition (i.e., condition in the absence of any conspecifics) was inhibited to a greater extent for dominant individuals than for submissive individuals (Stamm 1961). While this echoed contemporary research on humans at the time, which was evidencing complex social facilitation and inhibition effects (e.g., Guerin and Innes 1982; Zajonc 1965), similar efforts to examine and understand these phenomena in non-human animals, and primates more specifically, have been almost non-existent up until very recently.

On the one hand, the rise of cognitive research being conducted in zoos has led more researchers to examine the influence of cognitive testing (e.g., Herrelko et al. 2012; Hopper et al. 2016; Ruby and Buchanan-Smith 2015) and visitors’ presence on primates welfare (Carder and Semple 2008; Roth and Cords 2020; Wood 1998). This work has revealed important inter-species as well as intra-species differences. For instance western lowland gorillas (*Gorilla gorilla gorilla*) from one group displayed more anxious behaviours as the number of visitors increased while no such association was observed in a different group (Carder and Semple 2008).

Besides welfare indices, the visitors’ presence could also influence primates’ cognitive performance itself. Surprisingly, this question has rarely been addressed despite important potential theoretical and research practice implications. From this limited number of currently available studies, one study conducted in a zoo context demonstrated that when completing an attentional touch-screen task, Japanese macaques (M*acaca fuscata*) showed lower accuracy but faster reaction times as the number of zoo visitors increased (Huskisson et al. 2021). This would suggest that the visitors might have distracted the monkeys when they were paying attention to the task, a phenomenon that is also observed in humans (Baron 1986). However, the fact that the monkeys showed faster reaction times does not completely align with the presence of visitors capturing their attentional processes. Instead, the authors suggested a stress-related explanation where the juvenile monkeys (i.e., most of the individuals tested in this study) might have experienced some stress as the size of the crowd increased and sped up their responses (Cronin et al. 2018). It is indeed possible that an increasing number of visitors might be perceived as a threat for this small sized species. However, with this interpretation in mind, little is known about whether similar effects on cognitive performance would be observed in larger sized species such as chimpanzees. Indeed, very recent analyses (published after the pre-registration of the present research) of a large data set from a university research institute have indicated that chimpanzees’ accuracy performance on a working memory task was not influenced by the presence and size of an unfamiliar and unknown audience (e.g., humans visiting the facilities), whereas such performance was affected by the presence and number of experimenters (Lin et al. 2024). Nevertheless, data with chimpanzees living in a zoo setting, who potentially experience the presence of an unfamiliar audience to a greater extent than conspecifics living a university research institute facility, is still lacking. Answering this question would be particularly important for both evolutionary and research purposes as chimpanzees share a last common ancestor with humans and are currently the most represented primate species in comparative cognitive research (Hopper 2017).

On the other hand, another important aspect from zoo settings relates to the presence of conspecifics (Ruby and Buchanan-Smith 2015), and yet again, very little is known regarding the effects of their presence on cognitive performance in primates. The limited work so far published has focused on monkey species and observed opposite effects depending on the cognitive demands of the task. For instance, faster latencies were observed in the presence of conspecifics on simple cognitive tasks (e.g., touching the same stimulus on a screen across several trials; e.g., Monfardini et al. 2016; Reynaud et al. 2015) but longer response times were found on harder cognitive tasks such as a conflict attentional task (Huguet et al. 2014). Additionally, these studies also reported further modulations through social factors such as hierarchical rank (i.e., stronger detrimental effects in the presence of a more dominant individual) and affiliative relationships between individuals (i.e., greater facilitation in the presence of a closer individual). However, it is unclear how these influences would vary for species such as chimpanzees, who experience less stable hierarchical structure and social relationships with their conspecifics (de Waal 2007) than the monkey species previously tested (see also Gullstrand et al. 2024; Kaigaishi and Yamamoto 2024). Examining these conspecifics’ presence effects on chimpanzees can also provideimportant data regarding the underlying factors of audience effects in humans such as reputation management, self-image perception and normative aspects (Bond 1982). For instance, a previous study comparing task performance between human children and chimpanzees found that the two species retrieved more rewards when competing with another conspecific than when being observed and in the presence of another conspecific (Engelmann et al. 2016). However, human children, but not chimpanzees, showed greater performance in the observed condition (i.e., where a conspecific was positioned in front of the participant, without completing the task) as compared to the mere presence condition, (i.e., where the other individual was not in the same room, but visible to the participant). These differences between the two species in terms of how conspecifics influence decision-making might potentially lead to dissimilar conspecifics’ presence effects on cognition (see also Nettle et al. 2013). Finally and more broadly, investigating conspecifics' presence effects can also provide important insights regarding the influence of specific research practices that differ between research contexts (e.g., primates always tested in isolation, in the presence of particular conspecifics, or potentially with all group members present).

In the present study, we therefore examined the effects of the presence of visitors and conspecifics on chimpanzee’s working memory. This research was conducted at the Budongo Research Unit (BRU) in Edinburgh Zoo (UK) where chimpanzees can voluntarily, and without separation from their conspecifics, take part in cognitive research. Their participation can be seen by the public visiting the zoo as part of the overall visitors’ experience, thus providing a unique opportunity to examine the questions addressed by this study.

To this aim, we tested chimpanzees’ cognitive performance using the Rotating Grid Task (adapted from Reindl et al. 2021). In this task, chimpanzees were presented with a rectangular 4 × 4 grid containing eight boxes (four in the inner centre and four in the outer corners). The four boxes in the outer corners were used as potential hide places for reward (i.e., a grape). In each trial, one of these boxes was baited and chimpanzees had to identify and remember the correct location of the reward after a retention interval of 8 s. During this interval, the box was either not rotated or was rotated 90° or 180° (clockwise). This task assessed the ability to generate a mental representation of the location of the reward, store it and (where there was a rotation during the retention interval) to manipulate it to correspond to the correct updated location at the end of the trial. Therefore, different trial types in this task required different loads of working memory processing to successfully store and monitor the correct location of the reward (e.g., Ebert et al. 2025; Hyun and Luck 2007; Lehmann et al. 2014). A similar task has been used in a previous study with chimpanzees, whereby the experimenter first baited the grid box, then increased the working memory demands by introducing a secondary and interfering task during the retention interval (Völter et al. 2022). However, here, we used the rotation manipulation from Reindl et al. (2021) because it allows for multiple levels of difficulty within the same task, providing a signature of internal validity. While the chimpanzees were completing the task, we collected observations about the presence and activity of the visitors as well as about the presence and approximate physical distance of the conspecifics in the testing area.

In contrast to previous work (at the time of the pre-registration), our study tested a different species (chimpanzees) and used only accuracy data as is commonly done with working memory tasks. Therefore, in our pre-registration, we had only one confirmatory hypothesis (Hypothesis 1) and four explorative hypotheses (Hypotheses 2, 3, 4 and 5).

To begin with the confirmatory hypothesis on chimpanzees’ overall performance, we expected their performance to be worse on trials with more degrees of rotation than on trials with less rotations (H1). Moving on to effects of the visitors’ presence hypotheses, we initially predicted that higher numbers of visitors would lead to a decrease of chimpanzees’ accuracy (Huskisson et al. 2021). However, as our final data did not contain enough variation in crowd size (see Data Analyses section), this hypothesis (H2) was therefore restricted to the presence and absence of visitors (not pre-registered as such). Specifically, we predicted that the chimpanzees’ performance to be worse in the presence than in the absence of visitors (H2). Additionally, we predicted that chimpanzees’ performance would be worse when visitors were active than passive (H3). Regarding the conspecifics’ presence, based on previous research on baboons (*Papio papio*) (Huguet et al. 2014), we predicted that chimpanzees would be less accurate in the presence of conspecifics, especially if they are closer (in terms of physical distance) to them (H4). Finally, we predicted that if the visitors’ and/or conspecifics’ presence and activity or distance did indeed decrease performance, these effects should be stronger for harder trials (more attention-demanding) than for easier trials (less attention-demanding; H5).

## Methods

### Participants

Our participants were chimpanzees housed at the Budongo Research Unit (BRU) which operates within the Royal Zoological Society of Scotland’s Edinburgh Zoo (UK). These chimpanzees live in a natural group with access to both indoor and outdoor space with vegetation and artificial structures and are provided with food and enrichment throughout the day. On most days, they can voluntarily take part in non-invasive cognitive testing, which takes place in a dedicated testing area with water available ad libitum and in view of the visitors. Chimpanzees are never food or water deprived, nor separated for research purposes.

The group of chimpanzees comprised 15 individuals at the start of this study (20th June 2024) but this number dropped to 13 very early during our data collection after the passing of two individuals (from 20th August 2024). 11 individuals started the training sessions but only 8 passed the desired criteria or were willing to engage with the apparatus and were further tested during the test sessions (see Table 1 for the full demographic details).

This research was approved the School Ethics Committee of the School of Psychology and Neuroscience at the University of St Andrews (PS17835/PS17836) as well as the Scientific Committee at the BRU at Edinburgh Zoo.

**Table 1:** Demographic details, number of trials and mean correct responses of each chimpanzee participant (*n* = 8)

ID	Sex	Age (in years)	Number of trials for conspecifics analyses (*N* = 955)*	Number of trials for visitor analyses (*N* = 857)**	Mean correct responses (binomial test)
Eva	F	43.5	72	37	0.65 (*p* <.001)
Frek	M	30.7	148	148	0.76 (*p* <.001)
Kilimi	F	31.3	94	94	0.72 (*p* <.001)
Liberius	M	25.4	120	120	0.31 (*p* =.086)
Masindi	F	4.4	174	116	0.60 (*p* <.001)
Paul	M	31.3	51	50	0.35 (*p* =.065)
Qafzeh	M	32.2	139	135	0.32 (*p* =.030)
Velu	M	10.0	157	157	0.66 (*p* <.001)

*excluding experimental errors (e.g., wrong rotation)

**excluding data with observations done every 60 s, no video and no clock

## Materials

**Fig. 1:**
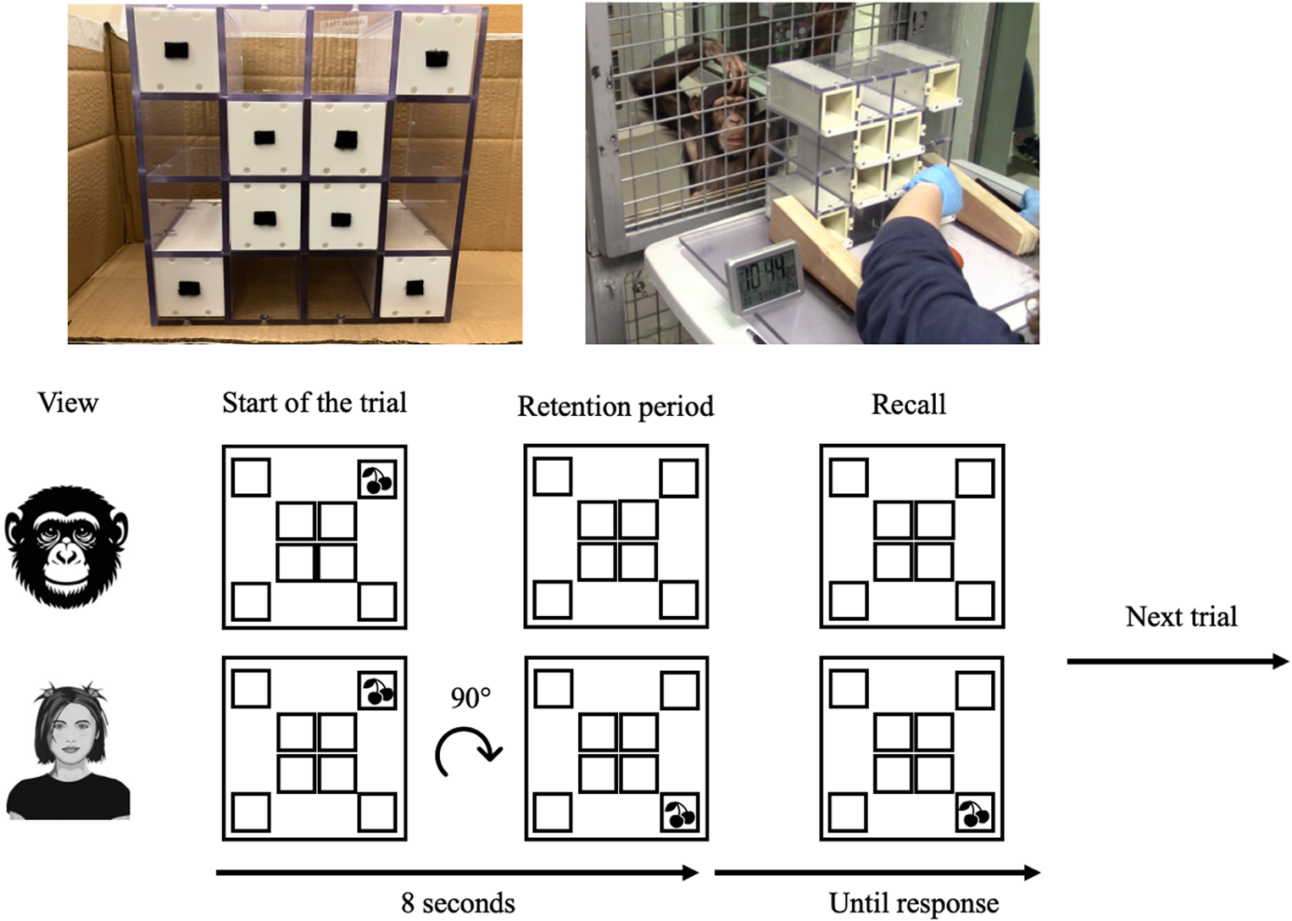
Photos of the grid used in this study from the chimpanzees’ view (top left) and of one trial during a testing session from the researcher’s view (top right). Below is a schematic representation of a 90° rotation trial from both the chimpanzees’ and researcher’s views

Chimpanzees’ cognitive performance was assessed using the Rotating Grid Task, which is a non-verbal task assessing mental rotation and working memory capacities (Reindl et al. 2021; Völter et al. 2022). As shown in Fig. 1 (top left picture), the materials consisted of a 4 × 4 Plexiglas grid (27.5 cm x 27.5 cm and 14 cm depth) containing eight small storage boxes (6.1 cm x 6.1 cm, 14 cm depth) that were positioned in the four corners (Outer) and in the middle square (Inner) compartments of the grid. These boxes were possible hiding locations for the food reward (i.e., a grape) on each trial. The grid was positioned on a table with a sliding support that could be moved forward to be within reach of the chimpanzees (Fig. 1; top right picture). On the table, a digital clock was visible in the video recordings and was used in the coding to match each trial with the closest observation (based on the time stamp available on Google Form).

## Procedure

The data collection started on 20th June 2024 and ended on 28th November 2024 and occurred on both weekdays and weekends (depending on researchers’ availabilities). Each research session lasted about 4 h from 9am to 1pm. During this time, chimpanzees were free to come and engage with the Rotating Grid Task. Only one research station dedicated for this study was set up during all sessions, although some research sessions were shared with another researcher so two stations (one for each separate study) were available to the chimpanzees.

**Fig. 2:**
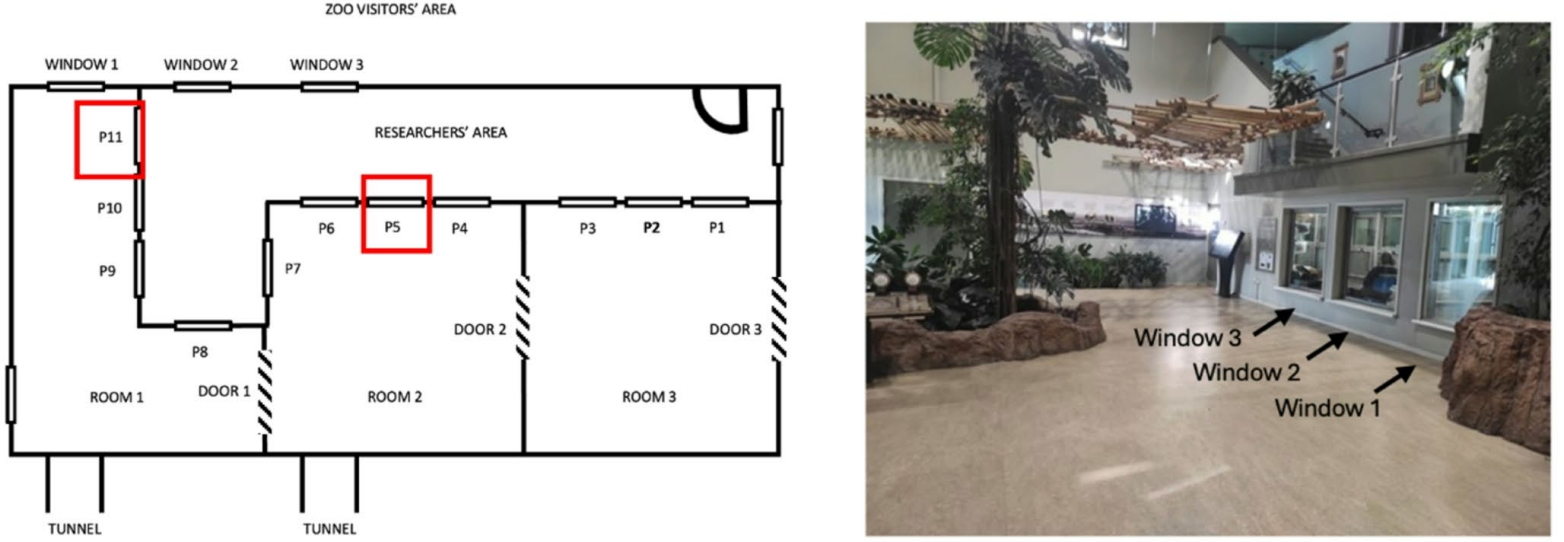
Schematic illustration of the testing area at the BRU and photo of the visitor area with the view on the three different windows

The sessions took place either at the research station panel 11 (P11; closest to one of the visitors’ windows) or panel 5 (P5; which is more central and allows for more opportunities to exit the research area; see schematic representation of the testing area in Fig. 2). The choice to set up the research station at P5 was made to increase participation of individuals who seemed less comfortable at P11 where exit options are more limited (see Fig. 2). Indeed, after two months of data collection, only 4 individuals (Eva, Masindi, Kilimi and Frek) were regular participants. Including P5 as a testing location in some sessions allowed us to gain additional 4 regular participants (Velu, Paul, Liberius and Qafzeh). However, testing at P5 was very occasional and did not exceed 20% of the total trials included in our analyses (see Table S3 in our Supplemental Material for more details).

Chimpanzees performed training and test blocks consisting of 10 and 12 trials, respectively. They were able to begin a session, leave and come back within a block for a maximum of 12 trials per research session. However, on some instances, chimpanzees completed more trials during one research session; for instance, if only 7 trials were completed the previous session, they had an opportunity to finish that block and start a new block. There were three different experimenters in total conducting the data collection of the test trials (three females, K did 59%, E did 27% and C did 14% of the test trials). The experimenter was the same within a day, but their identity varied between days according to their availabilities. One observer (one male) conducted all the observations on visitors.

## Training trials

Chimpanzees were first given two sessions of ten training trials (with no rotation) where only one box with the food reward was in the grid. On these trials, the box was placed in one of the four Outer compartments whereas for another ten trials, this box was placed in one of the four Inner compartments. Once the food reward was placed inside the box, the apparatus was moved forward so the chimpanzees could push the box to indicate their response (see Fig. 1). On these sessions, 9 chimpanzees passed the desired criteria (6/10) for both compartments (*mean* = 0.92, *sd* = 0.12). Two chimpanzees (Lucy and Louis) failed this training as they only pushed the boxes in the bottom Outer compartments regardless of the correct location. These successful 9 chimpanzees moved onto the next training phase, which consisted of at least one further session (maximum of 3 per Compartment version) of ten training trials where all the boxes were in the grid and the box containing the reward was placed in one of the Outer compartments only (see Table S1 in Supplemental Material). 7 chimpanzees (Frek, Kilimi, Liberius, Masindi, Paul, Qafzeh, Velu) achieved the minimal score (mean = 0.8, sd = 0.15). 1 chimpanzee (Eva) was incorrectly given the full Outer compartments test trials (including trials with rotation and occlusion; see next section) instead of another Outer compartment training trials. She nevertheless achieved the minimal score during these full Outer compartments test trials (score of 0.6) and therefore continued with testing. Another individual (Edith) failed to achieve the minimal score on the Outer compartment training trials (score of 0.5). A total of 8 chimpanzees were considered as having passed the training trials and were given test trials.

## Test trials

The first chimpanzees to complete training sessions (Eva and Masindi) were given the initial full test trials (on both the Inner and Outer compartments) as given in Reindl et al. (2021). This included three Retrieval trials, three Updating trials and four Manipulation trials. The Retrieval trials involved placing the food reward in one of the Outer compartments and moving the apparatus towards the chimpanzees without any rotation and occlusion (similar to the training trials). The Updating trials were trials where after placing the box back into the apparatus, the experimenter rotated the box clockwise with 90**°**, 180**°** or 270**°**. The Manipulation trials involved 0**°**, 90**°**, 180**°** or 270**°** rotation but the view on the boxes (i.e., the internal structure of the grid) was occluded with a plastic cover during the rotation. For the Manipulation trials with 0° rotation, the view of the boxes was occluded for about 5 s to match with the time it was occluded on trials with rotation degrees. Each time a food reward was placed in the targeted box and then placed back in the Inner or Outer compartment, the experimenter waited 18 s before allowing the chimpanzees to give their responses (i.e., by pushing the apparatus forward). This ensured that all trials were of the same duration regardless of the degree rotation. Eva completed three of these sessions with a mean score of 0.2 and Masindi completed two of these sessions with a mean score of 0.3. As their performance was very low (see Table S2 in Supplemental Material) and their willingness to participate dropped, we modified the procedure. Specifically, we only used the Retrieval trials (0° rotation) as well as the 90° and 180° rotation Updating trials. The overall duration of the trial was reduced from 18 s to 8 s and kept it constant for all trials. Chimpanzees were given a maximum of 13 sessions containing 12 trials each (with four 0° rotation, four 90° rotation and four 180°) that were given in pseudo-randomised order. All chimpanzees performed significantly above chance level, although this difference was only marginal for Liberius and Qafzeh (Table 1).

### Presence and distance of the conspecifics

After each trial, the experimenter recorded whether other conspecifics were present or not in the testing area. More specifically, this area is divided into three rooms (1, 2 and 3; see Fig. 2), and the testing sessions occurred either in room 1 (at P11 in Fig. 1and 83% of the 955 trials kept in the analyses) or in room 2 (at P5 in Fig. 2 and 17% of the remaining data points; see Table S3). When the chimpanzees were tested in room 1, the experimenter coded whether the conspecifics were in the same room (1; close), in room 2 (medium) or in room 3 (far), or 0 if no conspecifics were present (alone). When the chimpanzees were tested in room 2, the same coding system was used, but this time the physical distance of the conspecifics was considered close if in that same room (2) or medium if they were in rooms 1 and 3. In the case that the conspecifics were present in several rooms, we kept only the closest room in which they were as the measure of the physical distance of these conspecifics for that trial. Importantly, the doors connecting the research area and the chimpanzees’ main enclosure were always kept open, so the chimpanzees were free to access any of the rooms at any time.

## Presence and activity of the zoo visitors

While the chimpanzees were completing the Rotating Grid Task, one observer was collecting observations on the zoo visitors who were present near the windows that look into the experimenter’s and chimpanzees’ side (Fig. 1). The observer recorded how many zoo visitors were in front of these windows, what actions they were doing, what noise they were making, and whether or not they were looking at the chimpanzees. To capture these behaviours, we developed an ethogram (see Table 2) based on two previous studies that have observed zoo visitors (Huskisson et al. 2021; Roth and Cords 2020) and on two 2-hour observational sessions conducted in the visitor area before the start of the data collection (12th and 19th June 2024).

The observations were collected using a scan sampling technique focusing on the whole group of zoo visitors entering the area visible from the chimpanzees’ view. However, for the activity level, we focused on the activity that was displayed closest to the chimpanzees (focusing on Windows 1 and 2 when testing occurred at P11 or on Windows 2 and 3 when testing occurred at P5; see Fig. 2). If multiple zoo visitors were in front of the windows and several were active, we recorded the activity that happened closest to the chimpanzee being tested. If some visitors were active and other passive at any of the two target windows, we recorded the activity of the active visitors using the same logic (i.e., closest to the chimpanzee if possible). A similar rule applied for the variable noise and orientation. For instance, if at least one visitor was gazing at the chimpanzee being tested at any of the two relevant windows, the orientation was coded as towards the chimpanzee. We used a time sampling method to record these behaviours, and recordings began as soon as the first box was baited. We initially recorded the observations every 60 s but after 5 sessions, we used a shorter interval of 30 s to avoid having only one observation for two consecutive trials (the 5 first sessions were discarded in the analyses regarding the visitors’ effects, see Data Analyses). That is, every 30 s, a vibrate alert on a beeping device indicated to the observer to record the variables of interest on a smartphone using a Google Form (that also recorded a time stamp for each observation). Each observation was matched with a trial based on the closest match between the time displayed on the clock when the experimenter put the baited box in the apparatus and the time stamp on the Google Sheets. A total of 857 trials were kept for these analyses (see Table 1 and Data Analyses section for details of trial exclusion) and again 82% of these trials took place at P11 and the remaining 18% at P5.

**Table 2:** Ethogram for zoo visitors’ presence, activity level, gaze orientation and noise level

Number of Visitors
0. None
1. Very low (1–10)
2. Low (11–20)
3. Medium (21–40)
4. Large (above 41)
**Activity Level**
0. No movement
1. Active
a. Hand raising (pointing, taking a picture, lifting child)
b. Hand clapping
c. Walking
d. Running, jumping
e. Fighting, banging, throwing something
f. Leaning towards the window
g. N/A (no visitors at the two windows)
**Looking at/Orientation towards**
a. Chimpanzees
b. Others
c. Not sure
d. N/A (no visitors at the two windows)
**Noise Level**
a. Silent
b. Whispering
c. Talking at a normal level
d. Talking with a raised voice
e. Yelling, screaming
f. N/A (no visitors at the two windows)

### Data analyses

The analysis plan was pre-registered on the Open Science Framework (https://osf.io/5knjm) prior to any data analyses and the procedure below followed this pre-registration unless explicitly stated otherwise.

The analyses were performed using R version 4.3.1 (R Core Team, 2023). We used a Generalised Linear Mixed Modelling (GLMM) approach to analyse the data as it allows us to focus on each individual trial (instead of averaged trials) and to add random effects accounting for within-subject variabilities (Muradoglu et al. 2023).

Our data consisted of accuracy data that was binary: 0 (for incorrect responses) and 1 (for correct responses), we therefore used Binomial Logistic GLMMs. As pre-registered, our analyses strategy started by fitting a null model with a simple random structure (1|ChimpanzeeID) that was compared to full and reduced models. This null model was entered as:

$$ \begin{aligned}&~glmer(Accuracy{\text{ }}\left( {1/0} \right){\text{ }}\sim {\text{ }}1{\text{ }} + ~\left( {1|ChimpanzeeID} \right),\\ &~family{\text{ }} = {\text{ }}binomial\left( {link = `logit`} \right) \end{aligned}$$

We then fitted the full model that included the visitors or conspecifics variable of interest and its interaction with the task difficulty. We also fitted the main model with only the main effects of the visitors/conspecifics variable and the task difficulty variable. Once these models were fitted, we checked whether the addition of the main effects (without and with their interaction) provided a better fit to the data than the null model using the function *anova()* and also whether there was any overdispersion and collinearity issues in the main and full GLMMs using the package *performance*. To test the significance of the main effects, we compared the full model with the corresponding reduced models that lacked the predictor of interest using likelihood ratio tests. This nested model comparison method allows for testing whether the interaction term significantly adds explanatory power or not. If not, it can be dropped when testing the main effects, reducing the chances of misleading results due to a lack of power and of type I error resulting from multiple testing. This was done using the *drop1()* function. Odds ratios (not pre-registered) were used for effect sizes and following the interpretation given by Chen et al. (2010), effect sizes with odd ratios lower or between 1.68 and 3.47 were considered as small, between 3.47 and 6.71 as medium and above 6.71 as large. Pairwise comparisons were conducted using the package *emmeans* (Lenth et al., 2024). We also computed Bayes Factors (BFs) to weight the evidence for the null and alternative hypothesis (not pre-registered) using the packages *brms* (Bürkner 2017). This was done by comparing the different reduced, main and full GLMM models fit with a Bernoulli family. More precisely, to compute the BFs , we compared a model that included the specific term(s) of interest against an otherwise identical model that excluded only that particular term (e.g., a model without a specific main effect, or a model without a specific interaction). To report the BF_10_, we set the Cauchy distribution with location 0 and scale 1/√2 as a prior distribution for a coefficient parameter (Rouder et al. 2009). To interpret the BFs, we followed Lee and Wagenmakers (2014) with a BF_10_ of 1 regarded as ‘no evidence’ in favour of either the alternative or null hypothesis (model), BF_10_ between 1 and 3 as ‘weak evidence’, between 3 and 10 as ‘moderate evidence’, between 10 and 30 as ‘strong evidence’, 30–100 as ‘very strong evidence’ and over 100 as ‘decisive evidence’ for the alternative hypothesis or model. In contrast, BF_10_ of 1/3–1 is viewed of ‘weak evidence’, 1/10–1/3 as ‘moderate evidence’, 1/30–1/10 as ‘strong evidence’, 1/100–1/30 as ‘very strong evidence’ and under 1/100 as decisive evidence for the null hypothesis.

Finally, plots were generated using the *ggplot2* package (Wickham et al.,^ 2023^).

Chimpanzees completed a total of 966 trials. From these trials, 11 trials were removed due to experimental errors (e.g., wrong rotation) and so 955 trials were used for our analyses on the conspecifics’ presence. For the analyses on the visitors’ presence, we only kept trials where the visitors’ observations were collected every 30 s but not every 60 s. We also removed trials where the video recordings did not work, and it was therefore not possible to link these trials to the corresponding visitors’ observations. All in all, 98 trials were removed, and 857 trials were kept for our analyses on the visitors’ presence (see Table 1). For the visitors’ activity, we further excluded trials where no visitors were present (a total of 425 trials were removed) to strictly compare trials where visitors were active or not, when they were present.

We initially planned to analyse the effects of the number of visitors on chimpanzee’s working memory performance. However, we noted that there were very rare occurrences of Large (*n* = 2) and Medium (*n* = 7) number of visitors (see Table S4). We therefore merged these different variables into a more general variable accounting for the presence of visitors (yes or no) and we are reporting this non-pre-registered analysis. Similarly, we planned on analysing the effects of the noise level and gaze orientation of the visitors on chimpanzees’ cognitive performance. But again, we noted very rare instances of Loud (*n* = 6), Raised Voice (*n* = 28) and Whispering (*n* = 41) for the Noise Level, and of Others (*n* = 9) for gaze orientation. As these numbers further decreased when splitting across rotation levels and chimpanzee individuals, these two visitor behaviours were not further analysed (see Tables S4 to S8 for details on the number of trials as function of the different levels of visitors’ presence and activity and conspecifics’ distance and presence).

Note that (i) the repeated main effect of rotation found in the different analyses is a redundant statistic using the same performance data (on 955 trials for the analyses on the presence of conspecifics and on 857 trials for the analyses on the presence of visitors) on subject accuracy and (ii) there was no main effect of the session number (*p* =.133) indicating that chimpanzees did not get significantly better with time.

## Results

### Presence of visitors

The main and full models were significantly better than the null model, χ2 = 46.94, *p* <.001, and, χ2 = 47.63, *p* <.001. These models showed no overdispersion (*p* =.720 and *p* =.696, respectively) and no collinearity between the predictors (VIFs = 1.00).

**Fig. 3:**
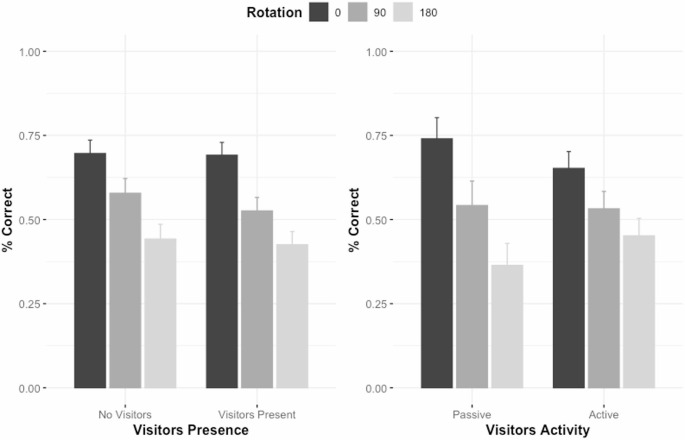
Accuracy in percentage as a function of rotation (0°, 90° and 180°) and the presence of visitors (no visitors, visitors present; left) and the activity of the visitors (active, passive; right). Accuracy on the hardest trials (180° rotation) was always significantly above chance (one-sided binomial test, *ps* < 0.001)

On accuracy level, there were main effects of rotation, χ2 = 46.15, *p* <.001, BF_10_ = 5.84 × 10^7^ (decisive evidence for H1), but not of the presence of visitors, χ2 = 0.52, *p* =.471, OR = 1.12, BF_10_ = 0.22 (moderate evidence for H0), with no interaction between these factors, χ2 = 0.69, *p* =.710, BF_10_ = 0.16 (moderate evidence for H0; see Fig. 3; left).

### Activity of the visitors

The main and full models were significantly better than the null model, χ2 = 23.69, *p* <.001, and, χ2 = 24.56, *p* <.001. These models showed no overdispersion (*p* =.552 and *p* =.584, respectively) and no collinearity between the predictors (VIFs = 1.00).

There were main effects of rotation, χ2 = 22.89, *p* <.001, BF_10_ = 1071.27 (decisive evidence for H1), but not of the activity of the visitors, χ2 = 1.04, *p* =.307, OR = 1.26, BF_10_ = 0.38 (weak evidence for H0), with no interaction between these factors, χ2 = 0.86, *p* =.649, BF_10_ = 0.21 (moderate evidence for H0) on chimpanzees’ accuracy (see Fig. 3; right) .

### Presence of conspecifics

Regarding the presence of conspecifics, the main and full models were significantly better than the null model, χ2 = 60.91, *p* <.001, and, χ2 = 62.07, *p* <.001. These models showed no overdispersion (*p* =.856 and *p* =.792, respectively) and no collinearity between the predictors (VIFs = 1.00).

**Fig. 4:**
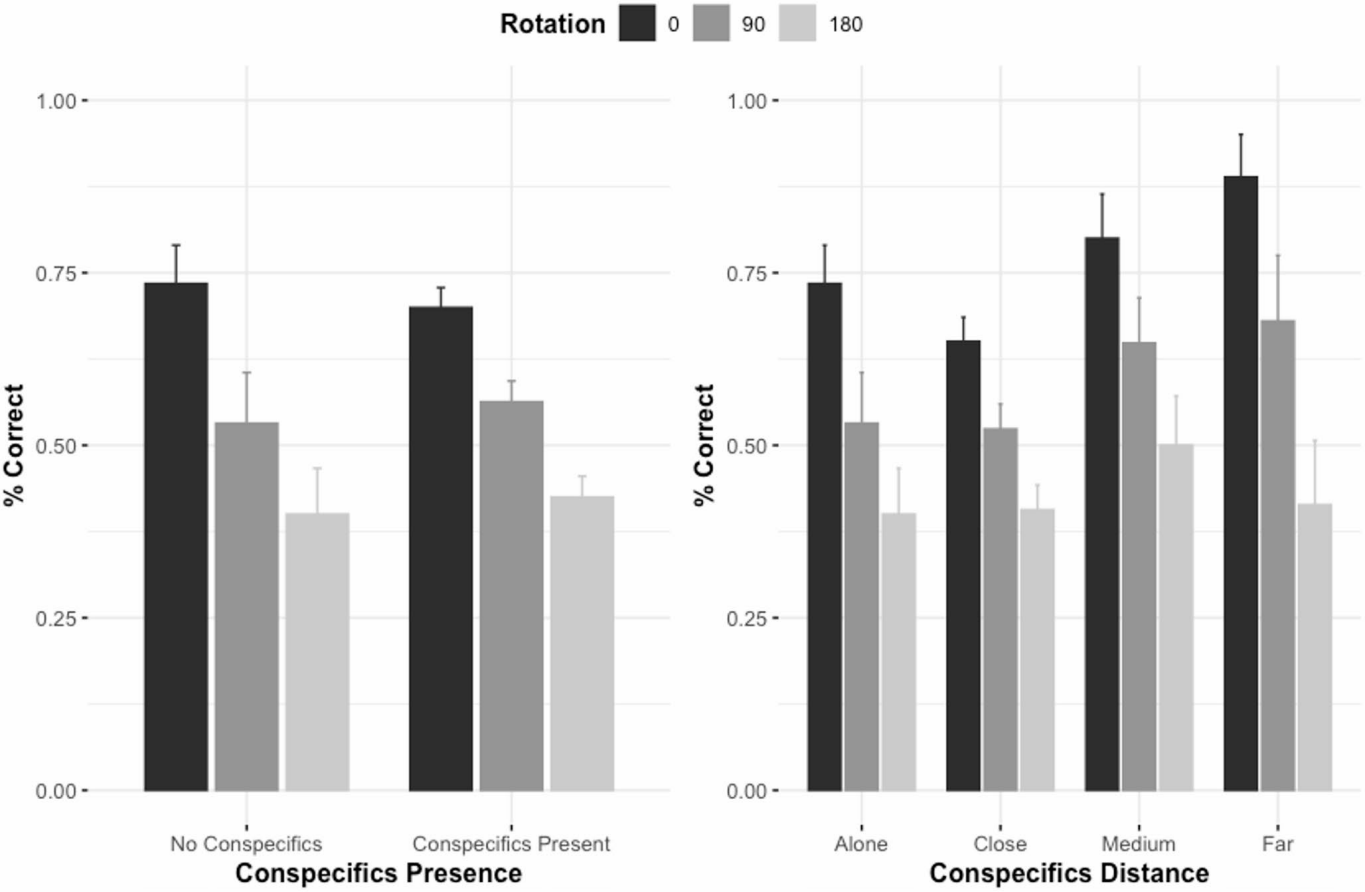
Accuracy in percentage as a function of rotation (0°, 90° and 180°) and the presence of conspecifics (no conspecifics, conspecifics present; left) and the distance of the conspecifics (alone, close, medium, far; right). Accuracy on the hardest trials (180° rotation) was always significantly above chance (one-sided binomial test, ps < .001).

There were main effects of rotation, χ2 = 60.71, *p* <.001, BF_10_ = 6.26 × 10^10^ (decisive evidence for H1), but not of the presence of conspecifics, χ2 = 0.56, *p* =.453, OR = 0.86, BF_10_ = 0.27 (moderate evidence for H0), with no interaction between these factors, χ2 = 1.16., *p* =.561, BF_10_ = 0.15 (moderate evidence for H0) on chimpanzees’ accuracy performance (Fig. 4; left).

### Distance of the conspecifics

Regarding the distance of conspecifics, the main and full models were significantly better than the null model, χ2 = 63.27, p < .001, and, χ2 = 67.10, p < .001, and 

 showed no overdispersion (*p* =.912 and *p* =.888, respectively) and no collinearity between the predictors (VIFs = 1.01). Again, there were main effects of rotation, χ2 = 61.33, *p* <.001, OR = BF_10_ = 8.60 × 10^10^ decisivev evidence for H1), but not of the distance of the conspecifics, χ2 = 2.92, *p* =.403, BF_10_ = 0.04 (strong evidence for H0), with no interaction between these factors, χ2 = 3.82., *p* =.700, BF_10_ = 0.01 (very strong evidence for H0; Fig. 4; right). 

## Discussion

In this study, we examined the effects of the presence of zoo visitors (and their activity level when present) and of conspecifics on chimpanzees' performance while they completed a cognitive task requiring them to engage working memory.

First of all, consistent with our confirmatory hypothesis regarding task difficulty effects on performance (H1), we found that in all analyses, performance of the chimpanzees on the Rotation Grid Task was worse on harder trials (i.e., with more rotation degrees) than on easier trials (i.e., with less rotation degrees). This indicates that our version of the Rotating Grid Task is appropriate to test different levels of chimpanzee’s working memory and that chimpanzees were indeed engaging more cognitive effort while solving the task. Moreover, another important finding was the lack of interaction between the different degrees of rotation and all the visitors' and conspecifics' variables, revealing that chimpanzees’ working memory decreased as the degrees of rotation increased regardless of potential external social presence factors. These results strongly speak in favour of the internal validity of the Rotating Grid Task (i.e., more degrees taps into more controlled processes), which has previously been shown in children (Reindl et al. 2021), and which is evidenced here in the present study in chimpanzees.

Regarding our main research questions, we predicted that the presence of visitors would decrease chimpanzee’s performance (H2), that active visitors would be more impactful than passive visitors (H3) and that these effects would occur more on harder than easier trials (H5). However, none of these predictions were confirmed by our data as no significant effects of the visitors’ presence and activity on chimpanzees’ scores were found, regardless of the difficulty of the trials. These non-significant effects were further supported by Bayes Factors giving support for the null hypotheses in all analyses. These results are contrary to what has been observed on a simple computerised attentional task in Japanese macaques, where a negative effect on accuracy level was observed as the number of zoo visitors increased (Huskisson et al. 2021). But, our results are in line with other research showing that the presence and the number of zoo visitors as well as university research institute visitors do not influence chimpanzees’ foraging behaviours and working memory performance, respectively (Hopper et al. 2015; Lin et al. 2024). Our results, coupled with the findings of Lin et al. (2024), suggest that chimpanzees might pay little attention to, and thus may not be distracted by, zoo visitors, or at least not to the point that this can be evidenced in accuracy data. However, in the analyses conducted by Lin et al. (2024), it was also found that chimpanzees’ accuracy performance was influenced by the number and familiarity of the experimenters. Specifically, chimpanzees were more accurate as the number of the experimenters increased when the task was highly cognitively demanding. Conversely, their accuracy performance decreased on less cognitively demanding versions of this task, particularly when the number of experimenters increased, and these latter were familiar to the chimpanzees.

Taken together, our present findings and the few available data suggest that several factors might determine whether and how chimpanzees are influenced by the presence of humans. Some of these important factors relate to the environment chimpanzees live in as well as to inter-individual differences, which likely result in important differences regardingr cognitive research experience as well asfamiliarity and relationships with humans (e.g., if physical contact is allowed or not between the researchers and the chimpanzees). Specifically, zoo-living chimpanzees have extensive experience of being observed by different unknown humans and may have learnt to ignore them over time. However, as mentioned above, a similar result was observed on chimpanzees housed in a university research institute (Lin et al. 2024). This seems to indicate that chimpanzees are not influenced by the presence of unknown humans in both settings tested provided they are used to having visitors. This said, it is worth mentioning that, in our study, all individuals who passed the training sessions are known to be regular participants whereas those who failed or did not finish these sessions tend to engage less with research (Kate Grounds, personal communication). Regular participants might therefore be more used to ignoring the presence of visitors, especially in the context of cognitive research, as compared those who participate less frequently. Consequently, the effects of the visitors’ presence could be greater on the latter group than the former group, although we are unable to make this conclusion for now. Conducting general group observations on how chimpanzees individually behave when visitors are present and are watching them (either outside and/or inside the experimental testing) would be valuable to address this point. For instance, it could be predicted that the individuals who generally avoid the presence of visitors might be the ones who are less likely to take part in research, especially when visitors are present.

Besides the absence of significant effects of the presence and activity of the zoo visitors, we also found no effects of the presence of conspecifics on chimpanzees’ performance on the working memory task. This effect was unchanged when we considered the physical distance between the chimpanzees and varying difficulty of the trials, and these non-significant effects were supported by Bayesian analyses, again giving support for the null hypotheses. These results are contrary to our predictions to find a decrease of accuracy in the presence of conspecifics (H4), especially for harder trials (H5). But it is also not in line with a previous study finding significant effects of the conspecifics presence in baboons’ attentional performance (Huguet et al. 2014). However, it is possible that our observations were not specific enough to capture these effects.

First, it could have been important to record whether the present conspecifics were passive (e.g., sitting and/or resting somewhere), active (conflicts, play), or making noise (e.g., displays, calls). Indeed, active conspecifics are likely to lead the participating individual to engage in more social monitoring (e.g., keeping track of the social interactions around to respond appropriately if needed, such as fleeing etc.), and potentially to devote less attention to the task at hand, ultimately deteriorating their task performance. In the same vein, it is possible that using the different rooms in the testing area to infer the distance between the chimpanzees might not have been accurate enough. Rather, future studies should also consider whether or not the true physical distance (in centimetres or meters) between conspecifics influences any effects resulting from that presence. Second, and equally important, which specific individuals are present around the participating chimpanzee should ideally be recorded, especially in light of previous studies showing that dominance status and friendships are key in modulating these effects (Huguet et al. 2014; Monfardini et al. 2017). Interestingly on this point, our data revealed that the two most dominant individuals (Liberius and Qafzeh) had the lowest rates of correct responses. Although speculative at this point, this could be because these dominant individuals need to devote greater attention to what is happening in the group, such as social interactions between particular conspecifics who are present, or calls produced by different group members out of sight (Stamm 1961). Again, while recording the identity of the conspecifics present is an important avenue for future research, it would also be essential to compute social factors such as hierarchical rank and/or Eigenvector centrality (i.e., how well individuals are connected between each other) through general group observations (see Gullstrand et al. 2024; Kaigaishi and Yamamoto 2024).Coupling experimental and observational data has a strong potential to unravel fine-grained effects of the presence and absence of conspecifics on chimpanzees’ cognitive performance, but also to shed light on how these effects vary in primates experiencing different levels of social tolerance in their group structure.

A final point regarding the effects of the conspecifics’ presence is that we could have expected those effects to be stronger than the effects of the visitors’ presence. Indeed, the presence of the former is likely to be both more causally and ecologically relevant than the presence of the latter. Indeed, contrary to the visitors, the conspecifics can directly and physically interact with the tested individual, and the nature of these interactions can depend on the differences in hierarchical status between individuals. The present data suggests no differences between the presence of social agents from the same species in the same room, or from a different species in a separate room, but future studies with more detailed measures of the conspecific' presence, more specifically, could tell a different story.

At a broader level, other factors might have contributed to the absence of significant effects reported here on both social presence variables. One of them is the overall duration of the trials. Indeed, when chimpanzees (Eva and Masindi) were first tested on the full version of the Rotating Grid Task, their participation quickly dropped after the first testing sessions. It is unclear whether it was because these trials were too long (18 s) or too difficult, or both. Nevertheless, it is possible that a longer duration could increase the potential interference caused by the presence of visitors and conspecifics. For instance, previous research on baboons has shown that social presence effects consuming executive resources are greater on trials with longer responses as top-down controlled processes take time to be deployed (Huguet et al. 2014). So, it is possible that the short duration of the trials (8 s) used in this study did not require chimpanzees to engage enough executive resources to abstractly maintain their representation of the correct location (Nelson & Narens, 1994), and led to lesser visitors' and conspecifics' presence effects on performance.

Another important variable to consider relates to inter-individual preferences regarding the task itself and the testing locations. Indeed, during our data collection phase, we observed that some chimpanzees were more willing to take part in research sessions if this involved an easy and familiar task with cups instead of a harder and less familiar task with the apparatus used for the Rotation Grid Task (although this information was not recorded). Similarly, some individuals also appeared more likely to participate if the research session occurred in the centre of the BRU testing area (P5 in Fig. 2), possibly because exit options were easier to access and/or because it was further away from the visitors’ window. Recall that testing initially occurred only at the station P11, but we decided to move the station to P5 as only 4 individuals were on the test sessions 2 months after the start of the data collection. Although our data revealed that all the chimpanzees who completed the test sessions did so more at P11 than at P5, there is little doubt that introducing the second location helped some of them to gain experience with the apparatus and further engage with the researchers. Thus, as for humans (Doebel 2020; Frick 2024), it is likely that chimpanzees’ performance on the Rotating Grid Task also strongly depends on individual preferences, and not only on their true abilities, which might also further shape how they are influenced by social presence.

Finally, some limitations of the present research should also be acknowledged. . The first relates to the sample size of our study, which was of only 8 individuals. Although this number almost reached the median number (*n* = 9) of participants in non-human primate studies in zoos (McEwen et al. 2022), achieving a higher sample size could reduce the weight of importance on inter-individual differences, commonly observed in comparative studies. One way to deal with this variability was to include the individuals in a random intercept, as well as using each trial collected on each individual (instead of averaging performance across all trials per individual), which increases the statistical power of the analyses (Muradoglu et al. 2023). The second limitation deals with the nature of the task and procedure we used, which produced only accuracy data. While social presence effects can be observed on this type of data (e.g., Huskisson et al. 2021; Lin et al. 2024), there is more evidence showing that these effects are stronger on latencies (in humans; see Garcia-Marques and Fernandes 2024). As such, it is possible that we would have observed some effects of both the visitors' and conspecifics’ presence if our task allowed the analyses of reaction time data. Moreover, we only recorded chimpanzees’ performance when they had already chosen to be near the visitors’ window. Given that some chimpanzees appeared to be more comfortable further away from the window closest to the visitors, it is possible that the effects of the visitors’ presence occurred at times outside of when we made our observations that we were unable to capture. Finally, we attempted to examine the effects of the number of visitors on chimpanzees’ performance. This was done by adapting ethograms used in previous studies (Cronin et al. 2018; Huskisson et al. 2021) and based on initial observations conducted in the visitor area. For instance, we reduced the number of zoo visitors that would fall into the very low number category from 1 to 20 to 1 to 10. However, we did not achieve a balanced number of observations between the different number of visitors categories, potentially because the majority of the data collection occurred after the summer holidays. As previous studies have reported significant effects of that number of individuals on primate cognition (Huskisson et al. 2021; Lin et al. 2024), this variable should be more carefully recorded in future similar studies (i.e., counting precisely the number of visitors present). Relatedly, it is important to note that even when no visitors were present, chimpanzees were still in the presence of at least one experimenter and one zookeeper and were never completing the task unobserved. Depending on feasibility, it would be valuable for future research to incorporate a ‘true alone’ condition when testing social presence effects in primates (Frick 2024).

To conclude, we report here that the presence and activity of zoo visitors (and similarly the presence of conspecifics, although we are cautious about this variable in our study) do not significantly influence chimpanzees’ performance on a working memory task. Importantly, given that these non-significant effects were found regardless of the difficulty of the degree of rotation and supported by Bayesian analyses, these results seem particularly robust. This has potentially important methodological implications for any cognitive research on great apes in zoo settings as the visitors may be present and watch the testing sessions. For instance, knowing that this presence has limited effects might encourage researchers to conduct research in zoo settings as well as zoos to welcome cognitive research in their facilities. The result also have implications such as benefiting science (and conservation) education if more research settings allow visitors to watch apes participating in research (Egelkamp and Ross 2019), and promoting cognitive research as a way to enhance enrichment and welfare in great apes (e.g., Herrelko et al. 2012; López-Álvarez et al. 2019). Future studies should attempt to confirm our present results and more thoroughly examine the effects of the presence of conspecifics on ape cognition.

## Supplementary Information

Below is the link to the electronic supplementary material.

Supplementary Material 1

## Data Availability

The data as well as the analytic code are available on the Open Science Framework at https://osf.io/gs8t2/?view_only=b32364ff0f3744e09eb74f7eac66fddf.
