# Supplementary material for: Chimpanzees’ working memory is not affected by the presence and activity of zoo visitors
Source: Anim Cogn. 2025 Dec 1;29(1):7. doi: 10.1007/s10071-025-02014-4 (PMC12774931; doi:10.1007/s10071-025-02014-4)
Supplement: Supplementary file 1 — Supplementary Material 1 [file 10071_2025_2014_MOESM1_ESM.docx]

**Supplemental Material: Frick, McEwen and Seed**

**Table S1: Number of trials and mean score (across all sessions) on training sessions as a function of compartment (outer, inner) and chimpanzee**

| ID | Outer Compartment Number of Trials and Mean of Performance | Inner Compartment Number of Trials and Mean of Performance |
| --- | --- | --- |
| Edith | 20 (0.75) | 10 (1) |
| Eva | 20 (0.70) | 30 (0.87) |
| Frek | 20 (0.95) | 20 (0.90) |
| Kilimi | 20 (0.70) | 20 (0.80) |
| Liberius | 20 (0.75) | 20 (0.70) |
| Louis | 30 (0.50) | 30 (0.40) |
| Lucy | 10 (1) |  |
| Masindi | 20 (0.95) | 20 (1) |
| Paul | 30 (0.63) | 20 (0.75) |
| Qafzeh | 28 (0.61) | 20 (0.65) |
| Velu | 20 (1) | 10 (1) |
|  |  |  |
| Total | 238 (0.75) | 200 (0.77) |

Two sample t-test: t = 0.32, df = 18.20, p = 0.750

**Table S2: Number of trials and mean score on full trial sessions as a function of compartment (outer, inner) and chimpanzee**

| ID | Outer Compartment Number of Trials and Mean of Performance | Inner Compartment Number of Trials and Mean of Performance |
| --- | --- | --- |
| Eva | 10 (0.60) | 20 (0) |
| Masindi | 10 (0.30) | 10 (0.30) |
|  |  |  |
| Total | 20 (0.45) | 30 (0.15) |

Two sample t-test: t = -1.41, df = 2, p = 0.293

**Table S3: Number of trials and mean score on test sessions as a function of testing location (Panel 5, Panel 11) and chimpanzee**

| ID | Panel 5  Number of Trials and Mean of Performance | Panel 11  Number of Trials and Mean of Performance |
| --- | --- | --- |
| Eva | - | 72 (0.65) |
| Frek | 36 (0.75) | 112 (0.77) |
| Kilimi | 28 (0.57) | 66 (0.79) |
| Liberius | 11 (0.36) | 109 (0.30) |
| Masindi | 30 (0.63) | 144 (0.59) |
| Paul | - | 51 (0.35) |
| Qafzeh | 24 (0.33) | 115 (0.32) |
| Velu | 33 (0.76) | 124 (63) |
|  |  |  |
| Total | 162 (0.61) | 793 (0.55) |

**Table S4: Number of trials on test sessions as a function of the level of visitors’ number and rotation degrees**

| Visitors Number / Rotation Degree | 0 | 90 | 180 |
| --- | --- | --- | --- |
| None | 135 | 128 | 129 |
| Very Low | 109 | 121 | 129 |
| Medium | 1 | 4 | 2 |
| Large | 1 | 0 | 1 |

**Table S5: Number of trials on test sessions as a function of the level of visitors’ presence and rotation degrees**

| Visitors Presence / Rotation Degree | 0 | 90 | 180 |
| --- | --- | --- | --- |
| Absence | 135 | 128 | 129 |
| Presence | 149 | 156 | 160 |

**Table S6: Number of trials on test sessions as a function of the level of visitors’ activity and rotation degrees**

| Visitors Activity / Rotation Degree | 0 | 90 | 180 |
| --- | --- | --- | --- |
| Passive | 50 | 48 | 55 |
| Active | 92 | 94 | 93 |

**Table S7: Number of trials on test sessions as a function of the level of conspecifics’ distance and rotation degrees**

| Conspecifics Distance / Rotation Degree | 0 | 90 | 180 |
| --- | --- | --- | --- |
| Close | 186 | 191 | 187 |
| Medium | 40 | 54 | 50 |
| Far | 27 | 25 | 29 |
| Alone | 64 | 47 | 55 |

**Table S8: Number of trials on test sessions as a function of the level of conspecifics’ distance and rotation degrees**

| Conspecifics Presence / Rotation Degree | 0 | 90 | 180 |
| --- | --- | --- | --- |
| Absence | 64 | 47 | 55 |
| Presence | 253 | 270 | 266 |
